# Association of ANKK1 polymorphism with antipsychotic‐induced hyperprolactinemia

**DOI:** 10.1002/hup.2737

**Published:** 2020-05-08

**Authors:** Olga Yu. Fedorenko, Diana Z. Paderina, Anton J. M. Loonen, Ivan V. Pozhidaev, Anastasiia S. Boiko, Elena G. Kornetova, Nikolay A. Bokhan, Bob Wilffert, Svetlana A. Ivanova

**Affiliations:** ^1^ Mental Health Research Institute Tomsk National Research Medical Center of Russian Academy of Sciences Tomsk Russia; ^2^ Division for Control and Diagnostics, School of Non‐Destructive Testing and Security National Research Tomsk Polytechnic University Tomsk Russia; ^3^ Department of Cytology and Genetics, National Research Tomsk State University Tomsk Russia; ^4^ PharmacoTherapy, ‐Epidemiology and ‐Economics, Groningen Research Institute of Pharmacy University of Groningen Groningen The Netherlands; ^5^ Policy Office for Quality and Innovation of Care (BZI), GGZ Westelijk Noord‐Brabant Halsteren The Netherlands; ^6^ Hospital, Siberian State Medical University Tomsk Russia; ^7^ Department of Psychotherapy and Psychological Counseling, National Research Tomsk State University Tomsk Russia; ^8^ Department of Psychiatry, Addictology and Psychotherapy, Siberian State Medical University Tomsk Russia; ^9^ Clinical Pharmacy and Pharmacology, University Medical Center Groningen, University of Groningen Groningen The Netherlands

**Keywords:** *ANKK1*, hyperprolactinemia, polymorphism, schizophrenia

## Abstract

**Objective:**

Schizophrenia is a severe highly heritable mental disorder. Genetic polymorphisms of dopaminergic pathways are related to pathogenesis of drug response. Hyperprolactinemia (HPRL), a common adverse effect of antipsychotics, is attributed to blockade of dopamine D2 receptors. Ankyrin Repeat and Kinase Domain containing 1 (*ANKK1*) gene is closely related to Dopamine Receptor D2 type (*DRD2*) gene functioning. We examined whether the functional polymorphism rs2734849 in the *ANKK1* gene is associated with antipsychotic‐induced HPRL.

**Methods:**

We recruited 446 patients with schizophrenia from among the Russian population of the Siberian region. The polymorphism rs2734849 in the *ANKK1* gene was genotyped with The MassARRAY® Analyzer 4 by Agena Bioscience™, using the kit SEQUENOM Consumables iPLEXGold 384. Genotype and allele frequencies were compared between groups of schizophrenia patients with and without HPRL using the χ^2^ test.

**Results:**

A comparison between schizophrenia patients with and without HPRL revealed significantly higher frequency of the C allele of the polymorphic variant rs2734849 in the *ANKK1* gene in patients with HPRL as compared to the patients without it (χ^2^ = 3.70; *p* = .05; odds ratio [OR] = 1.30 [0.99–1.69]).

**Conclusion:**

The functional polymorphism rs2734849 in the *ANKK1* gene was associated with HPRL in patients with schizophrenia.

## 1 INTRODUCTION

Schizophrenia is a severe mental disorder characterized by disruptions in thought processes, perceptions, volition, emotional responsiveness, cognitive processes, and social interactions. Antipsychotic drugs targeting dopamine neurotransmission are the principal mean of therapeutic intervention for schizophrenia (Rampino et al., 2019). Hyperprolactinemia (HPRL), a common adverse effect of antipsychotic drugs, is attributed to blockade of dopamine D2 receptors within the pituitary gland (Peuskens, Pani, Detraux, & De Hert, 2014).

Since genetic variation in gene coding for proteins that cross‐talk with *DRD2* at the molecular level are associated with response to antipsychotics (Rampino et al., 2019), the *ANKK1* gene (Ankyrin Repeat and Kinase Domain containing 1) is of particular interest. The encoded protein ANKK1, also known as protein kinase PKK2, belongs to a family of serine/threonine kinases involved in a number of signal transduction pathways, including cell proliferation, differentiation, and gene (including *DRD2* gene) transcription (Manning, Whyte, Martinez, Hunter, & Sudarsanam, 2002; Neville, Johnstone, & Walton, 2004; Ponce et al., 2009). The most frequently examined in a broad range of psychiatric disorders and personality traits functional polymorphism related to this gene is the Taq1A (rs1800497) (Dubertret et al., 2004; Fossella et al., 2006; Ponce et al., 2009; Suchanecka, Grzywacz, & Samochowiec, 2011; Kenny, Voren, & Johnson, 2013; Nymberg et al., 2014; Treble‐Barna et al., 2017; Persson & Stenfors, 2018). It was initially referred to DRD2 but later was shown to be mapped 10 kB downstream of *DRD2* in the neighboring gene *ANKK1* near the termination codon of the *DRD2* on chromosome 11q22‐q23 (Dubertret et al., 2010; Neville et al., 2004).

To date, there are inconsistent results from several association studies of *ANKK1* polymorphism with schizophrenia and its clinical phenotypes (Alfimova et al., 2017, 2018; Arab & Elhawary, 2015; Eisenstein et al., 2017; Nkam et al., 2017; Parsons et al., 2007; Ponce et al., 2009; Wishart et al., 2011; Yao, Pan, Ding, Pang, & Wang, 2015; Zhang et al., 2014). It has been hypothesized that rs1800497 polymorphism is likely to have a modifying effect rather than causative effect on schizophrenia (Zhang et al., 2014).

*DRD2/ANKK1* TaqIA allele status predicts striatal D2R specific binding in humans (Eisenstein et al., 2016; Gluskin & Mickey, 2016).

Interestingly, CYP2D6 metabolic activity affects mean antipsychotics daily dose only in the presence of *DRD2* Taq1A polymorphic allele. Subpopulation of schizophrenia inpatients with altered CYP2D6 activity (poor and ultra rapid metabolizers) and when carriers of Taq1A polymorphisms needs special attention of clinicians in aligning of antipsychotic treatment (Kurylev et al., 2018).

The preliminary epigenetic findings suggest that methylation levels at CpG site 387 of *ANKK1* may be associated with the treatment response to aripiprazole in patients with schizophrenia. Furthermore, methylation of *ANKK1* may affect dopaminergic neural transmission in the treatment of schizophrenia, and may influence treatment response (Miura et al., 2018).

Next to rs1800497 another functional polymorphism exists in *ANKK1* (i.e., rs2734849) which causes a nonsynonymous G to A transition leading to an amino‐acid change (arginine to histidine) in C‐terminal ankyrin repeat domain of *ANKK1* (Huang et al., 2009). There are far fewer data on the possible role of this polymorphism in psychiatric disorders. The exact role of SNP rs2734849 in *ANKK1* in psychiatric disorders and drug‐induced side effects has yet to be fully characterized.

Using the NF‐*κ*B‐luciferase reporter assay, Huang et al. (2009) found that the “A” allele of rs2734849 in *ANKK1* had greater suppression (∼30%) on NF‐*κ*B‐regulated luciferase activity than the “G” allele of rs2734849 indicating rs2734849 to be a functional polymorphism. In a protein sequence alignment and crystal structure comparison with a 12‐ankyrin repeat domain (Michaely, Tomchick, Machius, & Anderson, 2002), they found that the residue at 490 position resides on the surface of protein, a prerequisite for its involvement in mediating protein–protein interaction in the signal‐transduction processes leading to inhibition of NF‐*κ*B activity.

SNP rs2734849 in *ANKK1* is functional in yielding differential suppression of NF‐*κ*B‐regulated gene expression. Since transcription factor NF‐*κ*B is a necessary and sufficient signal factor to induce *DRD2* expression (Bontempi et al., 2007; Fiorentini et al., 2002), this suggests that variants of *ANKK1*, specifically rs2734849, may function to affect *DRD2* expression (Huang et al., 2009).

Since dopamine inhibits prolactin secretion by binding to D2 receptors expressed on the cell membrane of the anterior pituitary lactotrophs, we hypothesized that *ANKK1* polymorphism modulation of *DRD2* expression can be involved in the mechanisms of HPRL induced by antipsychotics due to their high affinity for the D2 receptor. In this study we investigated the association of *ANKK1* rs2734849 polymorphism with antipsychotic‐induced HPRL in Russian patients with schizophrenia.

## 2 MATERIALS AND METHODS

In this study, 446 patients (225 female/221 male) with schizophrenia were included from three psychiatric hospitals in Tomsk, Kemerovo, and Chita oblasts in Siberia, Russian Federation.

The study population and methods applied were previously described by Ivanova, Osmanova, Boiko, et al., 2017, Ivanova, Osmanova, Freidin, et al., 2017. Patients could participate if they had a clinical diagnosis of schizophrenia according to ICD‐10 (F20) and were between 18 and 75 years old. Exclusion criteria were a non‐Caucasian physical appearance (e.g., Mongoloid, Buryats, or Khakassians), pregnancy, relevant gynecological and endocrine (thyroid) disorders, organic brain disorders (e.g., epilepsy, Parkinson's disease) or relevant pharmacological withdrawal symptoms.

The study procedures were reviewed and approved by the Local Bioethics Committee of the Mental Health Research Institute. The study was recorded under protocol number N63/7.2014. Patients were included after providing written informed consent. None of the participants was compromised in their capacity to consent; therefore, consent from the next of kin was not recommended by the ethics committee. All works were carried out in accordance with The Code of Ethics of the World Medical Association (Declaration of Helsinki 1975, revised in Fortaleza, Brazil, 2013) for experiments involving humans.

Depending on the presence of HPRL (vide infra) this group of patients with schizophrenia was divided into two subgroups: 227 patients with HPRL and 219 patients without HPRL.

Patients were treated with conventional and/or atypical antipsychotic drugs in both oral and/or long‐acting formulations. One hundred ninety‐one patients received conventional antipsychotics. Haloperidol was the most commonly used conventional antipsychotic drug (110 patients). Other conventional antipsychotics included oral chlorpromazine (CPZ), chlorprothixene, trifluoperazin, and zuclopenthixol, and/or long‐acting formulations of haloperidol, zuclopenthixol, and flupenthixol. Atypical antipsychotics were used by 176 patients and included risperidone, clozapine, quetiapine, olanzapine, amisulpride, paliperidone, and sertindole. A total of 79 patients used various combinations of classical and atypical drugs. All dosages were converted into CPZ equivalents (CPZeq) to compare the antipsychotic exposure (Andreasen, Pressler, Nopoulos, Miller, & Ho, 2010).

### 2.1 Sampling

Blood samples for DNA extraction and prolactin determination were obtained after 8 hr of overnight fasting. For women of reproductive age, blood was, when possibly, taken in the follicular phase of the menstrual cycle. Blood samples for DNA extraction were collected in EDTA tubes and stored at −20°C until DNA isolation. Tubes with clot activator (CAT) were used to obtain serum for measuring prolactin concentrations (BD Vacutainer). The samples were centrifuged for 30 min at 1,500 rpm at 4°C to obtain serum.

### 2.2 Biochemical analysis

#### 2.2.1 Prolactin concentration

An accuBind ELISA Microwells kit (Monobind Inc., USA) was used to measure the prolactin concentration. The sensitivity of the used ELISA kit was 0.004 ng/well, what is equivalent to a sample prolactin concentration of 0.150 ng/ml. The upper limits for a normal prolactin concentration were set at ≤20 ng/ml for men and at ≤ 25 ng/ml for non‐pregnant, non‐nursing women. These criteria for HPRL were confirmed in literature (Kelly et al., 2013; Peuskens et al., 2014).

#### 2.2.2 DNA analysis

A standard phenol–chloroform micro method (as described by Ivanova et al., 2012) was used to isolate DNA from the leukocytes in the whole peripheral blood samples after pre‐freezing of the blood.

Genotyping for *ANKK1* rs2734849 was carried out on the MassARRAY® Analyzer 4 (Agena Bioscience™) using the set SEQUENOM Consumables iPLEX Gold 384. DNA sample preparation for SEQUENOM MassARRAY® Analyzer 4 includes several steps: a standard PCR reaction to obtain the amplification products, a shrimp alkaline phosphatase reaction to neutralize the unincorporated dNTPs in the amplification products, the PCR iPLEX Gold extension reaction, and then placing the samples on a special chip (SpectroCHIP Array) using NanoDispenser RS1000 prior to loading them into the analyzer.

### 2.3 Statistics

Data processing and analysis were performed in the *Statistical Package for Social Science*, version 20.0. The deviation from the Hardy–Weinberg equilibrium was tested with the χ^2^ test. To describe the results, frequency distribution tables were used for categorical variables. In the analysis of categorical variables, the *Fisher*'*s* exact test was used for the univariate analysis to evaluate the HPRL factors associated to the polymorphism. Differences were considered significant at *p* ≤ .05. The adjusted odds ratio (OR) with a respective 95% confidence interval (95% CI) was estimated.

## 3 RESULTS

### 3.1 Patients

We studied 446 Russian patients (225 female/221 male) with schizophrenia from the Siberian region (Tomsk, Kemerovo, and Chita oblasts). The baseline characteristics of the population are presented in Table 1.

**TABLE 1:** Demographic and clinical characteristics of patients with schizophrenia (*n* = 446)

Trait	All (446)	Male (221)	Female (225)	*p*‐value^a^
Age (mean ± SD)	42.1 ± 12.4	37.8 ± 11.9	45.2 ± 13.9	*p* < .0001
Duration of disease, years Median (IQR)	13 (6; 22)	11 (5; 18)	15 (7; 26)	*p* < .0001
Daily dose of antipsychotics in CPZeq Median (IQR)	425 (240; 750)	500 (300; 750)	372 (200; 750)	*p* = .003
Prolactin ng/ml Median (IQR)	23.32 (13.12; 49.96)	18.18 (10.61; 32.47)	34.89 (15.50; 65.03)	*p* < .0001

Abbreviations: CPZeq, chlorpromazine equivalents; IQR, interquartile range.

^a^ Calculated with either the *t*‐test or the Mann–Whitney *U* test, as appropriate.

The median dosage of antipsychotic drugs was 425 CPZ equivalents daily and the mean age of the patients was 41.5 years. In general, the mean duration of schizophrenia was 15 years. One hundred ninety‐one Patients were treated with conventional antipsychotics (42.8%), 176 patients with atypical antipsychotics (39.5%) and 79 with a combination of conventional and atypical antipsychotics (17.7%). Assuming a lower limit for the presence of HPRL at >25 ng/ml for females and >20 ng/ml for males (Kelly et al., 2013; Peuskens et al., 2014), 227 of the 446 patients suffered from HPRL and 219 patients were without HPRL.

### 3.2 Association of *ANKK1* rs2734849 with HPRL in the total group of patients with schizophrenia

Of a total group of 446 schizophrenia patients, genotyping results were obtained in 439 patients. The frequency of the rs2734849*С allele of the *ANKK1* gene in the total group was significantly higher in patients with HPRL compared to patients with normal level of prolactin (Table 2; χ^2^ = 3.7; *p* = 05; OR 1.30; 95%Cl: 0.99–1.69). When this group was divided by gender, the rs2734849*С allele of *ANKK1* was found to be a relative risk factor for antipsychotic‐induced HPRL in female patients only (Table 3; χ^2^ = 4.17; *p* = .04; OR 1.49; 95%Cl: 1.02–2.18). No statistically significant associations between *ANKK1* rs2734849 and HPRL were observed in the subgroup of male patients with schizophrenia (Table 4).

**TABLE 2:** Frequencies of genotypes and alleles of *ANKK1* rs2734849 in schizophrenia patients with and without HPRL (*n* = 439)

Genotypes, alleles	Patients with HPRL *n* (%)	Patients without HPRL *n* (%)	OR		χ2	*p*
			Value	95% CI		
СС	54 (24.3%)	40 (18.4%)	1.42	0.90–2.25	3.65	.16
CТ	110 (49.5%)	105 (48.4%)	1.05	0.72–1.52		
ТТ	58 (26.1%)	72 (33.2%)	0.71	0.47–1.08		
С	218 (49.1%)	185 (42.6%)	1.30	0.99–1.69	3.70	**.05**
Т	226 (50.9%)	249 (57.4%)	0.77	0.59–1.01		

Abbreviations: 95% confidence interval; ANKK1, Ankyrin Repeat and Kinase Domain containing 1; HPRL, Hyperprolactinemia; OR, odds ratio.

**TABLE 3:** Frequencies of genotypes and alleles of *ANKK1* rs2734849 in female schizophrenia patients with and without HPRL

Genotypes, alleles	Patients with HPRL, *n* (%)	Patients without HPRL, *n* (%)	OR		χ^2^	*p*
			Value	95% CI		
CC	31 (25.0)	14 (14.9)	1.90	0.95–3.83	4.43	.10
CT	64 (51.6)	49 (52.1)	0.98	0.57–1.68		
TT	29 (23.4)	31 (33.0)	0.62	0.34–1.13		
C	126 (50.8)	77 (41.0)	1.49	1.02–2.18	4.17	**.04**
T	122 (49.2)	111 (59.0)	0.67	0.46–0.98		

Abbreviations: 95% confidence interval; ANKK1, Ankyrin Repeat and Kinase Domain containing 1; HPRL, Hyperprolactinemia; OR, odds ratio.

**TABLE 4:** Frequencies of genotypes and alleles of *ANKK1* rs2734849 in male schizophrenia patients with and without HPRL

Genotype, alleles	Patients with HPRL *n* (%)	Patients without HPRL *n* (%)	OR		χ^2^	*р*
			Value	95% CI		
CC	23 (23.5)	26 (21.1)	1.14	0.61–2.16	0.39	.82
CT	46 (46.9)	56 (45.5)	1.06	0.62–1.80		
TT	29 (29.6)	41 (33.3)	0.84	0.47–1.49		
C	92 (46.9)	108 (43.9)	1.13	0.78–1.65	0.41	.52
T	104 (53.1)	138 (56.1)	0.88	0.61–1.29		

Abbreviations: 95% confidence interval; ANKK1, Ankyrin Repeat and Kinase Domain containing 1; HPRL, Hyperprolactinemia; OR, odds ratio.

The analysis of the distribution of prolactin levels depending on the *ANKK1* rs2734849 genotypes in patients with schizophrenia did not reveal any statistical differences although there was a tendency (*p* = .07) towards higher prolactin levels in patients with schizophrenia carrying CC and CT genotypes (Table 5).

**TABLE 5:** The distribution of prolactin levels depending on the *ANKK1* rs2734849 genotypes in patients with schizophrenia, (ng/ml)

Genotypes	N	Prolactin levels, me (Q1, Q3)	H	*р*
CC	94	25.12 (13.53; 46.12)	5.20	.07
CT	215	24.40 (13.96; 57.78)		
TT	130	18.36 (10.49; 44.73)

Abbreviation: ANKK1, Ankyrin Repeat and Kinase Domain containing 1.

### 3.3 Association of *ANKK1* rs2734849 with HPRL in the subgroup of schizophrenia patients treated with the risperidone/paliperidone

Due to the fact that, HPRL is significantly more common in patients receiving risperidone/paliperidone, the next step was the analysis in the subgroup of patients using the risperidone/paliperidone (*n* = 75).

No statistically significant associations between *ANKK1* rs2734849 and HPRL were found in the subgroup of patients with schizophrenia treated with the risperidone/paliperidone (Table 6).

**TABLE 6:** Analysis of association between HPRL and *ANKK1* rs2734849 in patients with schizophrenia from risperidone/paliperidone group (*n* = 75)

Genotype, alleles	Patients with HPRL *n* (%)	Patients without HPRL *n* (%)	OR		χ^2^	*р*
			Value	95% CI		
CC	14 (23.7)	3 (18.8)	1.35	0.34–5.42	2.58	.28
CT	28 (47.5)	5 (31.3)	1.99	0.61–6.43		
TT	17 (28.8)	8 (50.0)	0.40	0.13–1.25		
C	56 (47.5)	11 (34.4)	1.72	0.76–3.89	1.74	.19
T	62 (52.5)	21 (65.6)	0.58	0.26–1.31		

Abbreviations: 95% confidence interval; ANKK1, Ankyrin Repeat and Kinase Domain containing 1; HPRL, Hyperprolactinemia; OR, odds ratio.

## 4 DISCUSSION

A large number of individual genetic association studies have found that the TaqIA SNP in *ANKK1* is linked to psychiatric disorders (Ponce et al., 2009).

In this study, we investigated the association between functional polymorphism rs2734849 in *ANKK1* gene and antipsychotic‐induced HPRL in Russian patients with schizophrenia from the population of Siberia. For the first time we demonstrate that allele rs2734849*T of *ANKK1* gene mitigates the risk of antipsychotic‐induced HPRL in schizophrenia suggesting for a protective effect against developing this side effect, whereas carriage of the rs2734849*С allele was a relative risk factor for antipsychotic‐induced HPRL in schizophrenia. Our results support the involvement of *ANKK1* rs2734849 into pathogenesis of antipsychotic‐induced HPRL in schizophrenia, although the underlying molecular mechanisms are not clear.

*ANKK1* and *DRD2* genes belong to the same gene cluster, the NTAD cluster, an ancient cluster of which genes are apparently co‐regulated and may have emerged when the central nervous system became more complex (Feistauer, Vitolo, Campagnolo, Mattevi, & Almeida, 2018; Mota, Araujo‐Jnr, Paixão‐Côrtes, Bortolini, & Bau, 2012). A few in vitro studies with *ANKK1* gene mRNAs and proteins were able to show the potential connection between this gene and the dopaminergic system (Feistauer et al., 2018; Garrido et al., 2011; Hoenicka et al., 2007).

There are data about *ANKK1* rs2734849 to be functional in yielding differential suppression of NF‐*κ*B‐regulated gene expression. Since transcription factor NF‐*κ*B is a necessary and sufficient signal to induce *DRD2* expression (Bontempi et al., 2007; Fiorentini et al., 2002), variants of *ANKK1*, specifically rs2734849, may affect *DRD2* expression (Huang et al., 2009). These findings align with the results that *DRD2/ANKK1* gene polymorphisms alter the density of dopamine receptors (Stelzel, Basten, Montag, Reuter, & Fiebach, 2010).

It is known that dopamine holds a predominant role in the regulation of prolactin secretion. It directly inhibits the basally high‐secretory tone of the anterior pituitary lactotrophs by binding to D2 receptors expressed on their cell membrane resulting in a reduction of prolactin exocytosis. All typical antipsychotic medications are associated with sustained HPRL due to their high affinity for the D2 receptor and their slow dissociation from the receptor once bound, but atypicals differ quite dramatically in their propensity causing prolonged high prolactin levels (Fitzgerald & Dinan, 2008). As HPRL directly depends upon the functioning of DRD2 receptors on mammatrophic cells of the anterior pituitary gland (Peuskens et al., 2014), altered functionality of these receptors (particularly altered expression) is more likely to be observed.

Previously in the same sample of patients we studied a set of 41 SNPs of genes encoding the dopamine receptors DRD1, DRD2, DRD3, DRD4, the dopamine transporter SLC6A3, and dopamine catabolizing enzymes MAOA and MAOB (Fedorenko et al., 2017; Osmanova et al., 2019). No associations were found between the *DRD2* gene polymorphism and the antipsychotic‐induced HPRL in patients with schizophrenia. We have demonstrated the *MAOB* rs1799836 to be a potential risk factor for HPRL in men in the total group (Osmanova et al., 2019).

In the current study we hypothesize that in the total group of patients with schizophrenia those who carry rs2734849*С allele of *ANKK1* gene have higher density of D2 receptors on the anterior pituitary lactotrophs resulting in HPRL induced by antipsychotics due to their high affinity for the D2 receptors.

Risperidone is known to elevate prolactin due to its higher peripheral‐to‐central dopamine receptor potency (Fitzgerald & Dinan, 2008); Geers et al., 2020). Earlier in the same sample of patients we have found that the rs40184 and rs3863145 variants in *SLC6A3* gene appeared to be associated with HPRL in the subgroup of patients using the risperidone/paliperidone, but not with HPRL induced by other antipsychotic drugs (Osmanova et al., 2019). However, when only patients treated with risperidone/paliperidone were studied, no significant association was found with *ANKK1* rs2734849 polymorphism (Table 6). Another limitation of the study might be our reluctance to analyze genotyping data of *ANKK1* in combination with other genes. We genotyped a few other genes in much the same patient sample and in the same run (Geers et al., 2020; Ivanova, Osmanova, Freidin, et al., 2017; Osmanova et al., 2019), but this analysis would be insufficiently extensive and hypothesis‐driven. Our results extend previous findings about association of *ANKK1* with antipsychotic‐ induced side effects.

## 5 CONCLUSION

The results of this study indicate that the prevalence of antipsychotic‐induced HPRL in Russian patients with schizophrenia is higher in carriers of rs2734849*С allele of *ANKK1* gene whereas allele rs2734849*T of *ANKK1* gene has a protective effect against antipsychotic‐induced HPRL in this sample. Further investigation will be required to develop a better understanding of the mechanisms by which *ANKK1* gene polymorphism influence the drug‐induced side effects in schizophrenia.

## CONFLICTS OF INTEREST

The authors declare no conflicts of interest.
